# A Case of Application of Computer-Aided Design and Manufacturing Technology and Extended Reality Surgical Assistance to Marginal Mandibulectomy

**DOI:** 10.3390/jcm14010008

**Published:** 2024-12-24

**Authors:** Takahiro Nakada, Masahide Koyachi, Keisuke Sugahara, Akihiro Nishiyama, Mana Kawakami, Shintaro Nakajima, Kotaro Tachizawa, Kento Odaka, Satoru Matsunaga, Maki Sugimoto, Akira Katakura

**Affiliations:** 1 Department of Oral Pathobiological Science and Surgery, Tokyo Dental College, 2-9-18 Kandamisaki-cho, Chiyoda-ku, Tokyo 101-0061, Japan; tnakada@tdc.ac.jp (T.N.); koyachim@tdc.ac.jp (M.K.); nishiaki@tdc.ac.jp (A.N.); kawakamimana@tdc.ac.jp (M.K.); nakajimashintaro@tdc.ac.jp (S.N.); tachizawakotaro@tdc.ac.jp (K.T.); sgmt@med.teikyou.ac.jp (M.S.); katakura@tdc.ac.jp (A.K.); 2 Department of Oral and Maxillofacial Radiology, Tokyo Dental College, 2-9-18 Kandamisaki-cho, Chiyoda-ku, Tokyo 101-0061, Japan; odakakento@tdc.ac.jp; 3 Department of Anatomy, Tokyo Dental College, 2-9-18 Kandamisaki-cho, Chiyoda-ku, Tokyo 101-0061, Japan; matsuna@tdc.ac.jp; 4 Innovation Lab, Teikyo University Okinaga Research Institute, 2-16-1 Hirakawacho, Chiyoda-ku, Tokyo 102-0093, Japan

**Keywords:** extended reality, computer-aided designed and manufacturing, squamous cell carcinoma, image-guided surgery

## Abstract

**Background/Objectives:** Mandibular gingival squamous cell carcinoma (SCC) is the second most common oral cancer after tongue cancer. As these carcinomas often invade the mandible early, accurately defining the resection extent is important. This report highlights the use of preoperative virtual surgery data, computer-aided design and manufacturing (CAD/CAM) technology, surgical guidance, and extended reality (XR) support in achieving highly accurate marginal mandibulectomy without recurrence or metastasis. **Methods**: CT imaging data obtained a month before surgery were imported into Materialize Mimics and Materialize Magics (Materialize, Leuven, Belgium, Ver22.0) CAD/CAM software and used to design an osteotomy guide. An STL file was generated, and the guide was fabricated using a 3D printer (Objet 260 Connex; Stratasys Ltd., Eden Prairie, MN, USA) prior to the operation. An XR application, installed on a HoloLens (Microsoft, WA, USA) head-mounted display, projected a hologram onto the surgical field. **Results**: The rapid intraoperative diagnostic tests were negative, and histopathology confirmed SCC without vascular or perineural invasion. No complications, including occlusal or feeding problems and sensory abnormalities, were observed. Postoperative imaging 3 years later showed no recurrence. **Conclusions**: Combining CAD/CAM and XR techniques for mandibulectomy may improve surgical accuracy and safety in oral and maxillofacial surgeries, whereas in-house 3D printing aids in managing tumor progression.

## 1. Introduction

Mandibular gingival squamous cell carcinoma (SCC) is the second most common oral cancer, after tongue cancer. As most mandibular gingival carcinomas invade the mandible at a relatively early stage, accurately defining the extent of resection is important. In cases of mandibular gingival carcinoma, bone invasion should be considered, and resection of the surrounding soft tissues and jaw should be performed. While preserving as much tissue as possible is necessary to avoid esthetic and functional complications, radical cure of the lesion is important. In recent years, digital technology has been used to solve these problems and to improve the safety and accuracy of surgery. Among these, computer-aided design/computer-aided manufacturing (CAD/CAM) and extended reality (XR) technologies have been widely applied for surgical support in several medical specialties, and improvements in surgical accuracy and safety have been reported [1].

CAD/CAM and XR technologies are widely used in oral and maxillofacial surgery for jaw tumors [2] and orthognathic surgery [3]. Surgical guides fabricated using three-dimensional (3D) printers have improved surgical accuracy and safety and reduced surgical time [4]. In addition, surgical navigation using XR technology not only improves surgical accuracy and safety but also facilitates team communication by projecting preoperative medical imaging data onto the surgical field in 3D [5]. In the oral and maxillofacial region, where hard tissues, such as bone, are involved, the simultaneous use of these technologies can potentially improve surgical accuracy. In addition, although the osteotomy guide using CAD/CAM technology showed the extent of superficial resection and the angle of resection, there are few methods to check whether the guides are being used as planned during surgery, and there is room for improvement in the accuracy. Therefore, we decided to perform surgery while superimposing a hologram using the registration marker developed for the XR application. Herein, we report a case in which preoperative virtual surgery data, CAD/CAM technology, surgical guidance, and XR surgical support were used to perform highly accurate marginal mandibulectomy without recurrence or metastasis.

## 2. Materials and Methods

### 2.1. Case

**Figure 1:**
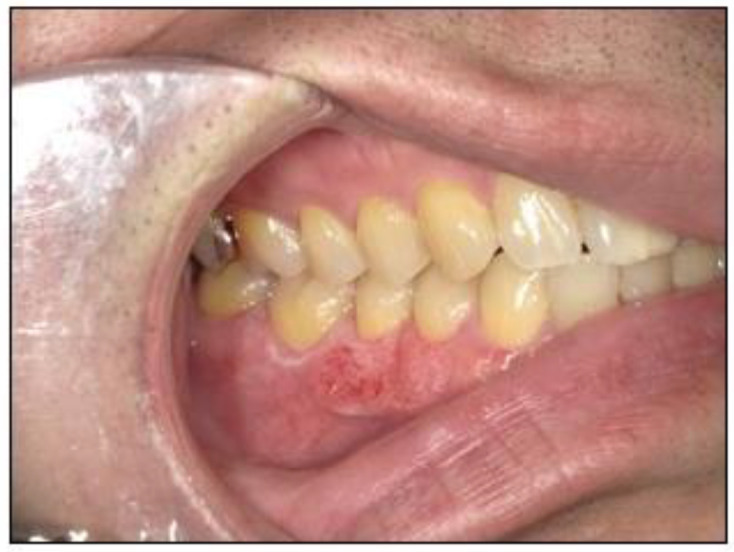
Preoperative intraoral photograph. Ulceration with a coarse surface, erythema, and white spots are seen from the interdental papilla between the mandibular right first and second molars to the center of the mandibular right first premolar.

**Figure 2:**
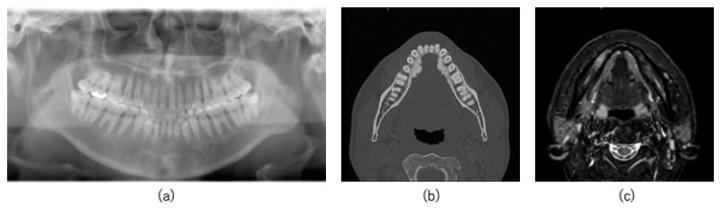
Preoperative imaging findings. (**a**) Preoperative panoramic radiograph. No significant bone destruction is observed. (**b**) Infiltration is observed in the cortical bone in the mandibular right second premolar region. (**c**) Short tau inversion recovery magnetic resonance imaging (MRI) displaying a high signal intensity area measuring 23 mm anteroposteriorly in the mandibular right first premolar to second molar region.

A 44-year-old man visited a local physician in February 2018 with a complaint of discomfort in the mandibular right buccal gingiva. The medical and family histories were unremarkable. In July 2019, the patient noticed a tendency for ulcer formation in the same area but was unable to visit the physician for 3 months owing to his work schedule. In 2020, he was referred to our hospital owing to persistent stomatitis in the mandibular right posterior region. Intraoral examination revealed a coarse gingival surface with erythema and white spots extending from the interdental papilla between the mandibular right first and second molars to the center of the mandibular right first premolar (Figure 1). Panoramic radiography showed no obvious bone destruction in the right mandible (Figure 2a). However, computed tomography (CT) revealed cortical bone infiltration in the mandibular right second premolar region (Figure 2b), and magnetic resonance imaging (MRI) revealed a 23 mm diameter high signal intensity area in the mandibular right posterior region (Figure 2c). Positron emission tomography/CT revealed no metastases. The biopsy results revealed SCC, and the patient was diagnosed with gingival carcinoma (T2N0M0) in the mandibular right posterior region. The treatment plan included a marginal mandibulectomy extending from the mandibular right canine to the mandibular right third molar with extraction of the corresponding teeth. An osteotomy guide was fabricated using CAD/CAM technology. Nevertheless, appropriate mandibular resection could not be confirmed intraoperatively.

### 2.2. Preoperative Preparation (CAD/CAM Osteotomy Guide, Virtual Surgery, and Fabrication of XR Application and Registration Marker)

**Figure 3:**
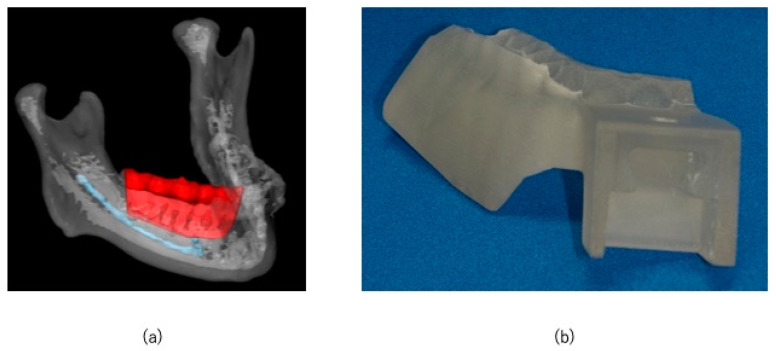
CAD/CAM-based osteotomy planning and guide. (**a**) The red area indicates the set resection area, and the mandibular canal is shown in blue; (**b**) osteotomy guide fabricated using CAD/CAM technology.

Three-dimensional images of the mandible were reconstructed with data from 0.6 mm thick slices using computed tomography (CT; Somatom Definition AS, Munich, Germany) scans. CT was performed within 1 month before surgery, and the data were imported into Materialize Mimics (Materialize, Leuven, Belgium) and Materialize Magics (Materialize, Leuven, Belgium) CAD/CAM software, and an osteotomy guide was designed. An STL file was created, and an osteotomy guide was fabricated using a 3D printer (Objet 260 Connex; Stratasys Ltd., Eden Prairie, MN, USA) 1 week before surgery. The modeling method was the polyjet system. The tumor resection margin was set at approximately 10 mm in the mesial and distal direction. The mesial and distal ends of the resection range were the extraction sockets of the mandibular right canine and third molar, respectively, and inferiorly, the resection was limited above the mandibular canal, where bone invasion had not reached. The resection line was determined to be connected to the buccal resection line on the mandibular canal above and on the lingual side. It is also designed to preserve the loop from the mandibular canal and the mental foramen (Figure 3a). An occlusal surface was added to the osteotomy guide to fix it to the upper and lower dentition (Figure 3b). Since the osteotomy guide is in a form that can be fitted in the mouth, it can be tested before surgery, and if there are any defects, it can be quickly re-created in the in-office laboratory. The unique markers and osteotomy guide used in this case are the same system used to reposition the maxilla during Le Fort I osteotomy, as we previously reported [3]. In that report, the percentage of bone measurement errors within 2 mm between the virtual operation and postoperative CT images averaged 90.3% of cases. In addition, the surgical guide can be tried on the patient before surgery, preventing fitting errors during surgery.

**Figure 4:**
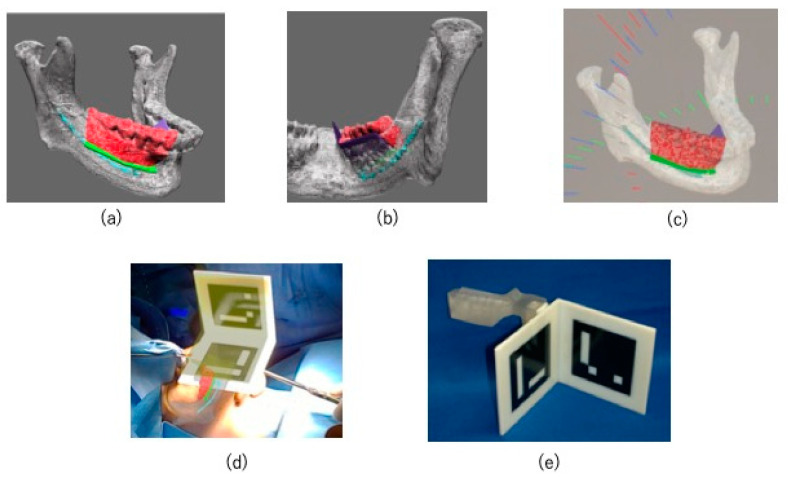
XR application design and implementation. (**a**,**b**) Application design. The area shown in red is the resection range, the area shown in light blue is the mandibular canal, and the areas shown in green and purple denote the resection angles; (**c**) hologram installed on HoloLens; (**d**) registration markers created using CAD/CAM. (**e**) The hologram was automatically superimposed on the surgical field in three dimensions using registration markers 149.

The CT DICOM data were converted into STL data for virtual surgery. In the absence of evidence of the lesion invading the mandibular canal, the preservation of the nerve loop extending forward from the mandibular canal and mental foramen was ensured. Although a high signal area was observed in this case on MRI, CT showed only slight bone invasion in the cortical bone. So, we thought it would be difficult to segment the exact tumor range. However, since we planned to use an osteotomy guide in this case, we thought it would be more effective to segment the resection line and display it using XR technology during marginal resection rather than segmenting the tumor itself. The resection area was shown in red, the mandibular canal was shown in light blue, and the resection angles were shown in green and purple (Figure 4a,b). An XR application was created from the STL data of the virtual operation using Holoeyes MD (Holoeyes, Japan).

The XR application was installed on a HoloLens (Microsoft, WA, USA), which is a see-through head-mounted display, to project a hologram onto the surgical field during surgery (Figure 4c).

The osteotomy guide was equipped with a connection to allow the attachment of registration markers developed by our department (Figure 4d) [6]. By incorporating the registration function into the XR application, the hologram could be automatically superimposed on the surgical field in 3D (Figure 4e). The hologram remained fixed to the superimposed spatial coordinates even after the registration markers were removed.

### 2.3. HoloLens Application

**Figure 5:**
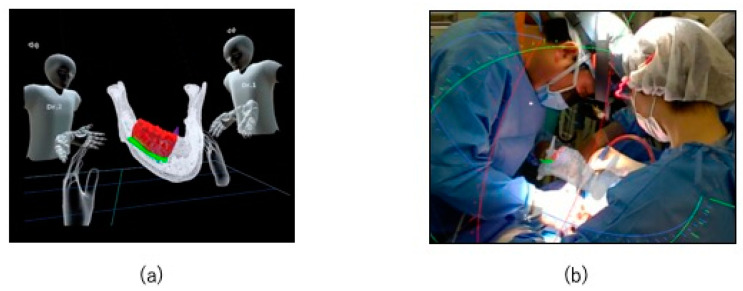
Preoperative discussion and intraoperative use of XR technology. (**a**) The holograms are shared among the surgeons and discussed preoperatively in the metaverse; (**b**) the surgeon operates while wearing the HoloLens.

The hologram was shared with the surgeons before surgery, and a preoperative discussion was held in the metaverse (Figure 5a). After wearing the HoloLens, hands were thoroughly cleansed before beginning surgery (Figure 5b).

### 2.4. Surgical Procedure

**Figure 6:**
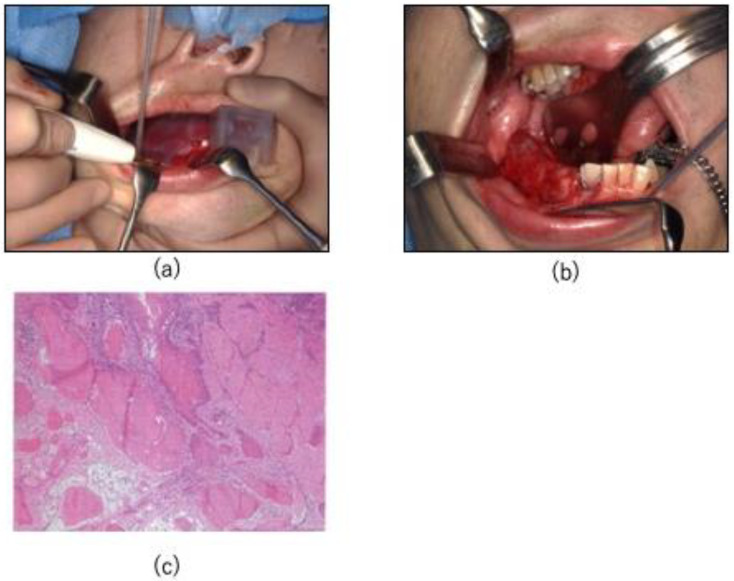
(**a**) Resection is performed using the osteotomy guide. (**b**) After resection, tie-over is performed, and a protective floor is attached. (**c**) All excised specimens have negative margins, and the histopathological diagnosis is squamous cell carcinoma, with no vascular or perineural invasion.

After registration of the surgical field and hologram, the markers were removed, and resection was performed using the osteotomy guide (Figure 6a). During resection, the hologram was checked in 3D to confirm that the resection was performed according to that in the virtual surgery and that the buccolingual plane was not shifted. A tie-over was performed after resection, and a protective floor was placed (Figure 6b). This protective floor was made of clear resin with a clasp. The surgery lasted for 2 h 29 min, and the amount of blood loss was 191 mL. The results of all the rapid intraoperative diagnostic tests were negative. The histopathological diagnosis was squamous cell carcinoma without vascular or perineural invasion (Figure 6c).

### 2.5. Evaluation

The superimposition of the preoperative and postoperative image data and evaluation was performed by GOM Inspect (GOM, Braunschweig, Germany).

Postoperative CT scans were taken under the same conditions as the preoperative scans. As a tie-over was performed during the operation, the scan was taken 1 month later, after the epithelialization of the wound.

## 3. Results

### 3.1. Surgical Accuracy

**Figure 7:**
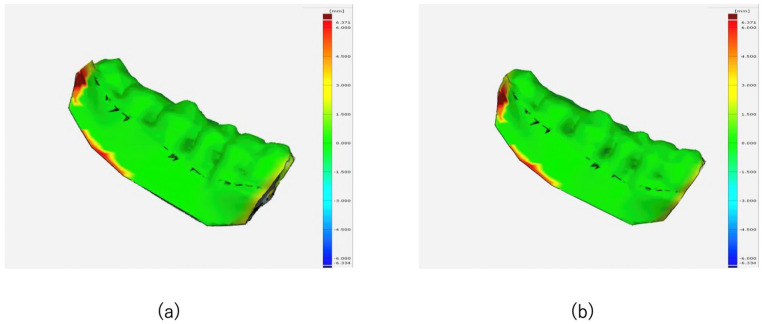
The bone surface error is measured by superimposing the 1-month postoperative CT image and the 3D image of the preoperative virtual surgery. (**a**) Bone surface error between Tv and T1 within 1 mm. (**b**) Bone surface error between Tv and T1 within 2 mm.

Surgical accuracy was evaluated by comparing the 3D image of the preoperative virtual surgery (Tv) and the 1-month postoperative CT image (T_1_) of the resected area using Materialize ^®^ Mimics. The bone surface error between Tv and T_1_ was measured, and it was ≤1 mm and ≤2 mm in 81.3% and 93% of the surface, respectively (Figure 7a,b).

### 3.2. Postoperative Evaluation

**Figure 8:**
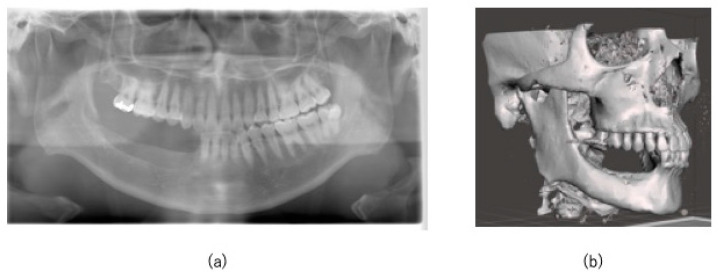
Postoperative 3-year follow-up imaging. (**a**,**b**) OPG and CT at the 3-year follow-up shows no recurrence and no sensory abnormalities in the inferior alveolar nerve.

No intra- or postoperative complications, including occlusal or feeding problems and sensory abnormalities of the inferior alveolar nerve, were observed, and no recurrence was observed on postoperative imaging 3 years after surgery (Figure 8a,b). A jaw prosthesis has been placed in the defect area, and the occlusion and feeding status are good.

## 4. Discussion

Osteotomy guides fabricated using CAD/CAM technology are useful for safe and efficient surgery. CAD/CAM technology has recently been used to improve surgical accuracy in head and neck surgery and reconstruction [2]. In addition to CAD/CAM technology, the use of XR technology in surgery has been reported in various fields in recent years [7]. Furthermore, cases of highly accurate mandibular reconstruction using iliac bone grafting in combination with CAD/CAM and XR techniques in patients with mandibular resection for the management of benign tumors have been reported [2].

Although conventional computer-assisted surgery allows the viewing of 3D images, the depth and size of the 3D structure tend to be incompletely understood because of the flat monitor. XR technology allows surgeons to intuitively understand the 3D position of the blood vessels and nerves. The XR system uses a head-mounted display that is less expensive and more portable than conventional navigation systems, making it easier to implement. In contrast, the setup of conventional navigation systems, including preoperative calibration, is time-consuming. Moreover, misalignment of the calibration during surgery may increase operation time [8]. In addition, while conventional AR-based navigation had the advantages of portability and low implementation costs, it was hard to overlay the surgical field and virtual objects with depth. This makes it difficult to provide accurate three-dimensional surgical guidance [9]. In this case, the combination of an osteotomy guide and XR technology using a proprietary registration marker allowed quick superimposition of preoperative virtual surgery data on the surgical field as a hologram [3]. The innovative aspect of this technique is that, because we approach the surgery from inside the mouth, we use our unique registration markers that match the dentition. This ensures that the surgical field and XR technology are superimposed.

CAD/CAM technology alone can guide the resection range; however, it cannot accurately guide the resection angle. In this study, using XR technology in combination with CAD/CAM allowed buccolingual resection while checking the resection angle and mandibular canal in 3D, which likely avoided damage to the mandibular canal. Postoperative accuracy evaluation revealed that 81.3% of errors were ≤1 mm. Considering the changes caused by postoperative bone resorption, we believe that the surgery was performed with high accuracy. There are many previous reports that have determined the extent and angle of resection [10,11]. But in this case, the aim was to further improve the accuracy of the resection angle and extent by using XR technology in combination. Recently, some CAD/CAM surgical guides for jaw reconstruction have included not only osteotomy lines but also dental implant guides [12]. This also has the advantage of allowing planned jaw prosthesis using implants. In this case, a removable prosthesis was used at the patient’s request.

The most notable feature of the new system is the surgical support for malignant tumors using both CAD/CAM and XR technologies. In conventional computer-assisted surgery, when a patient-specific implant is used, virtual surgery is performed based on CT data taken beforehand, and a custom-made plate is ordered and used according to the planned postoperative anatomical configuration of the patient [6,13,14]. The time from order placement to surgery is approximately 1 month. Therefore, responding to changes due to disease progression, as in the present case, may be impossible. In this case, no evidence of lesion progression was observed on the CT imaging; therefore, the surgical guide and application were not remade. However, even if the lesion had progressed, the surgical guide would have been made using an in-house 3D printer [15,16].

The use of XR technology allows the surgeon to view the preliminary virtual surgery data in 3D during surgery, which is useful for improving safety, educating inexperienced surgeons, and gaining a common understanding of the surgery. For example, using XR technology, surgeons can share preoperative information about the patient and repeat the procedure while interacting with each other. This enables simulations to be performed and improves the accuracy of the procedure [17,18,19]. XR technology also provides intuitive visual information to surgeons, which may improve their concentration and surgical safety [20].

This study had some limitations. Soft-tissue tumors are usually highly susceptible to intraoperative motion during the projection and superimposition of holograms, which can lead to loss of accuracy [21]. But in this case, the resection area included hard tissue, and the effects of intraoperative movement could be avoided. This enabled a stable surgical field and hologram superposition using XR technology. In this system, surgical accuracy is largely dependent on CAD/CAM, and XR technology is only used as a supplement. However, with the development and introduction of new hardware, more accurate surgery could be performed using XR technology alone [22]. CAD/CAM technology will likely continue to be utilized across various surgical domains in the future. Future studies are needed to address the limitations of CAD/CAM and XR technologies and to explore strategies for optimizing their application across various surgical domains.

## 5. Conclusions

The combination of CAD/CAM and XR techniques for mandibulectomy opens up the possibility of improving the surgical accuracy and safety for future oral and maxillofacial surgeries. Furthermore, the use of an in-house 3D printer helps compensate for tumor progression.

## Data Availability

This article data is unavailable due to privacy or ethical restrictions.
